# Comparison of Didactic Lectures and Activity-Based Learning for Teaching First-Professional MBBS Students in the Subject of Anatomy

**DOI:** 10.7759/cureus.51106

**Published:** 2023-12-26

**Authors:** Dewanshi Mishra, Shweta Singh, Abeer Z Khan, Sushobhana kumar, Pratibha Dwivedi

**Affiliations:** 1 Anatomy, Dr. Ram Manohar Lohia Institute of Medical Sciences (DRRMLIMS), Lucknow, IND; 2 Anatomy, Hind Institute of Medical Sciences, Barabanki, IND

**Keywords:** team-based learning (tbl), teaching-learning method, traditional teaching methods, activity-based learning (abl), didactic lectures

## Abstract

Background

In the field of medical education, traditional methods of teaching and learning have been used for a long time. Various new methods of learning, like activity-based learning (ABL), case-based discussion (CBD), and problem-based learning (PBL), are laying the foundation for this modern era of teaching. This study was a comparative study conducted to determine the effectiveness of activity-based learning and traditional lectures by teaching two topics to MBBS first-year professional students.

Aims

The study compares the effectiveness of didactic lectures and activity-based learning for MBBS first-year professional students in the subject of anatomy.

Methodology

The study was conducted in the Department of Anatomy Hind Institute of Medical Sciences, Barabanki, and included 100 MBBS (2022-2023) first-year students randomly assigned to Group A (n=50, 16 females and 34 males) and Group B (n=50, 23 females and 27 males) through chit methods. The participants were exposed to two different topics of anatomy in the form of activity-based learning as well as didactic lectures. Assessments were done immediately after the exposure in the form of a post-test, and results were analyzed for both groups.

Result

The results of the study showed that both activity-based learning and didactic lectures were effective in teaching the two topics, but the didactic lecture group had higher retention rates than the activity-based learning group. The mean score of post-tests of students who attended a didactic lecture on the brachial plexus (6.166± 2.11) was slightly higher than that obtained by students who attended activity-based learning (5.625 ± 2.12), but the p-value obtained was not significant (0.249). Whereas the mean of the scores of the post-test obtained by students who attended a didactic lecture on mammary gland was (8.45± 1.20), slightly lower than the mean of the scores of the post-test of students who attended activity-based learning on mammary gland (8.60± 1.16), but the p-value obtained was not significant (0.520).

Conclusion

This study provides evidence that didactic lectures play an important role in teaching anatomy to MBBS first-year students and cannot be replaced by activity-based learning alone. Lectures followed by activity-based learning can prove to be a newer and more effective teaching-learning method with better outcomes in the form of retention and conceptual understanding of the topics in anatomy.

## Introduction

Activity-based learning is an innovative approach to teaching that is becoming increasingly popular in medical education, especially in the field of anatomy. This teaching method involves engaging students in active, hands-on learning experiences that encourage them to explore and discover information for themselves. Activity-based learning is rooted in the idea that students are active learners rather than passive recipients of information [1]. If learners are provided the opportunity to explore on their own and are provided with an optimum learning environment, then learning becomes more joyful and long-lasting. In the first year of MBBS, where students are introduced to the foundational concepts of anatomy, activity-based learning can be an effective tool to help them develop a deeper understanding of the subject. The National Medical Commission has decreased the time allotted for didactic lectures and allotted more time for group discussions [2]. By providing opportunities for students to actively participate in the learning process, activity-based learning can enhance their retention of information, develop critical thinking skills, and promote a more holistic understanding of human anatomy.

The teaching of Phase 1 (preclinical phase) students included traditional teaching methods such as didactic lectures, textbook readings, etc. The learning strategies aim to enhance the learning process and engage students in analyzing and achieving higher levels of understanding of the subject matter [3,4].

In lectures, students are exposed to the topics through verbal explanations and visual aids, such as slides or diagrams, which may be difficult for students to conceptualize [5]. Textbook readings provide additional information and often serve as a reference for students to review later. Lab dissections involving students working in groups to dissect cadavers and examine anatomical structures first-hand are a good example of activity-based learning. The concept of didactic lectures has been used for many years. Some students may find them passive and unengaging, but when followed by an activity, they may prove to be a better option for creating interest in the subject and thus may give a better outcome.

According to a previous study, students have expressed a preference for lecture-based learning over case-based learning when it comes to preparing for written exams [6].

This study helped explore some of the key benefits of activity-based learning for MBBS first-year students and some methods to implement it effectively in the anatomy classroom.

Aim and objectives 

This study was undertaken to compare the effectiveness of didactic lectures as well as activity-based learning. The main objective was to calculate and compare the effectiveness of both teaching and learning methods with the help of post-test marks obtained by the students in a multiple-choice question-based assessment by teaching the same topic via didactic lecture and activity-based learning and to analyze students' perceptions of the two types of methods with the help of an open-ended questionnaire.

## Materials and methods

The study was a case-control analytical study conducted in the Department of Anatomy at Hind Institute of Medical Science, Barabanki, India. The study group consisted of 100 MBBS (2022-2023) first-year professional students. The age of the students ranged between 19 and 22 years. There were a total of 39 females and 61 males in the study group.

Ethical clearance

The study was conducted after getting approval from the Institutional Scientific Committee and Institutional Human Ethics Committee (HIMS/IHEC/2023/Faculty/Dr. Dewanshi Mishra /26/09/2023) with a waiver of consent.

Method

Hundred chits mentioning groups A and B were made (50 each). The age of the students ranged between 19 and 22 years. Students were asked to pick up the chits kept in a bowl and were randomly allocated into two groups, A and B, with each group consisting of 50 students. Group A consisted of 16 females and 34 males, whereas Group B consisted of 23 females and 27 males. Informed consent was obtained from the study participants. Two different sessions on the topics of brachial plexus and mammary gland were selected according to the currently scheduled timetable and planned according to the new competency-based medical education (CBME) curriculum.

Group A (n=50) received didactic lectures on the brachial plexus, while Group B (n=50) participated in an activity-based learning session for the same topic.

Conversely, for the next topic of the mammary gland, the two groups were shuffled, i.e., Group B (n=50) received didactic lectures on the mammary gland, while Group A (n=50) participated in an activity-based learning session for the same topic.

Standard lecture materials were used for the didactic lecture group, including chalk and board methods and PowerPoint presentations.

For the activity-based learning, 50 students were divided into 4-5 groups, with each group having 10-12 students, and an activity sheet consisting of questions to draw and label, enumerate, etc., was prepared for both topics. An additional activity using different colored wool, board pins, and thermocol sheets was conducted for the topic of the brachial plexus. In the activity for the brachial plexus, students were asked to make a model demonstrating the formation of roots, trunks, and cords of the brachial plexus with the help of wool and board pins.

**Figure 1:**
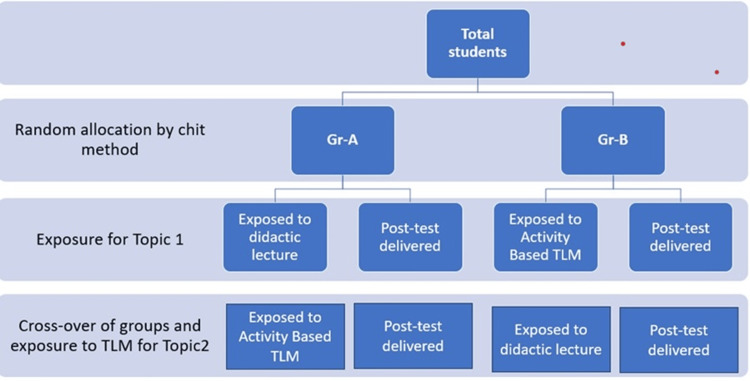
Flow chart showing the random allocation of subjects and the conduction of the two teaching-learning methods (TLM)

A post-test consisting of 10 multiple-choice questions via Google Forms was administered immediately after the intervention for the assessment, which was the same for both groups. The scores for the two topics of the mammary gland and brachial plexus (both lecture and activity group) were tabulated in a Microsoft Windows Excel sheet (Redmond, USA) separately. Students who were absent from any of the sessions and assessments were excluded from the study (Figure 1).

Statistical analysis

The statistical analysis was done using IBM Corp. Released 2015. IBM SPSS Statistics for Windows, Version 23.0. Armonk, NY: IBM Corp. Normality was checked using the Shapiro-Wilk test. The mean scores for both the didactic lecture group and activity group for both topics were calculated and compared, and the data was analyzed using the Mann-Whitney U test for significance.

A research hypothesis was made that there will be no change in the scores of students no matter which method of teaching is used.

## Results

The study was conducted over two weeks in the Department of Anatomy. The results of the study showed that both activity-based learning and didactic lectures were effective in teaching the two topics, but the didactic lecture group had higher retention rates than the activity-based learning group. The didactic lecture group also performed better on the post-test, but no statistical significance was found.

**Figure 2:**
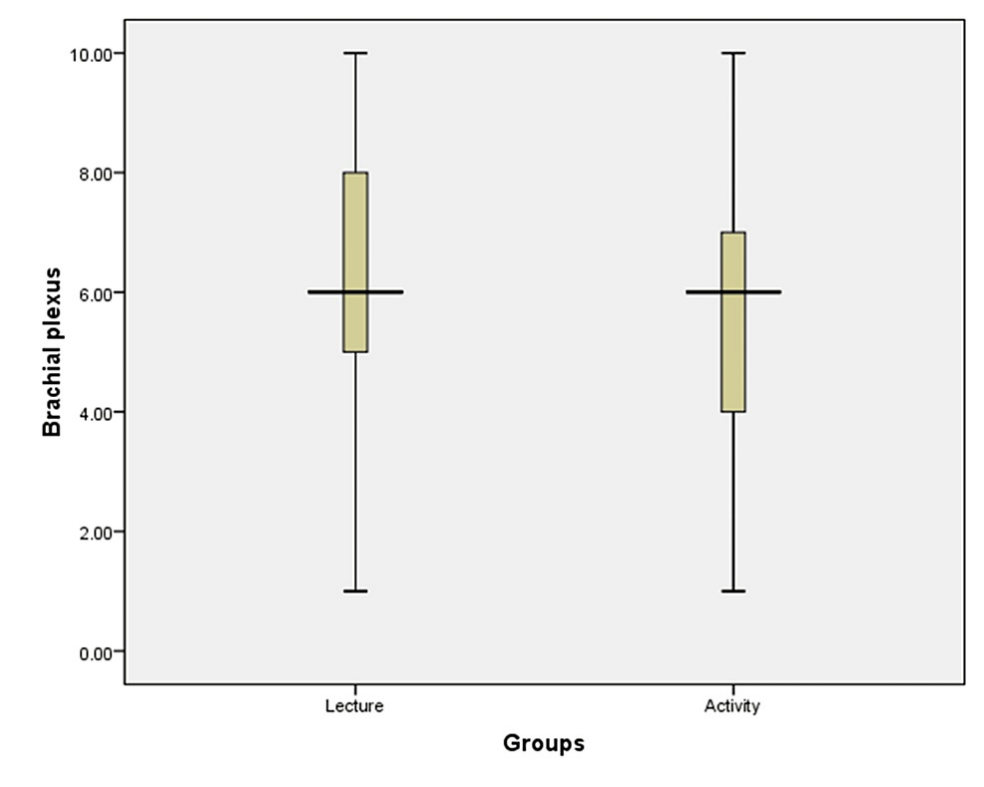
Box graph showing the comparison between the marks scored for the topic of brachial plexus taught by didactic lecture and activity-based learning methods.

The mean score of post-tests of students who attended (n=48) a didactic lecture on the brachial plexus (6.17) was slightly higher than that obtained by students who attended (n=48) activity-based learning (5.62), but the p-value obtained was not significant (0.249) (Table 1) (Figure 2).

**Table 1:** Comparison between the post-test marks of the two teaching and learning methods for the topic Brachial Plexus TLM: Teaching/learning materials

TLM	Mean ± SD
Lecture	6.17 ± 2.11
Activity	5.62 ±2.12
Total	5.90 ±2.12
p-value	p-value=0.249

**Figure 3:**
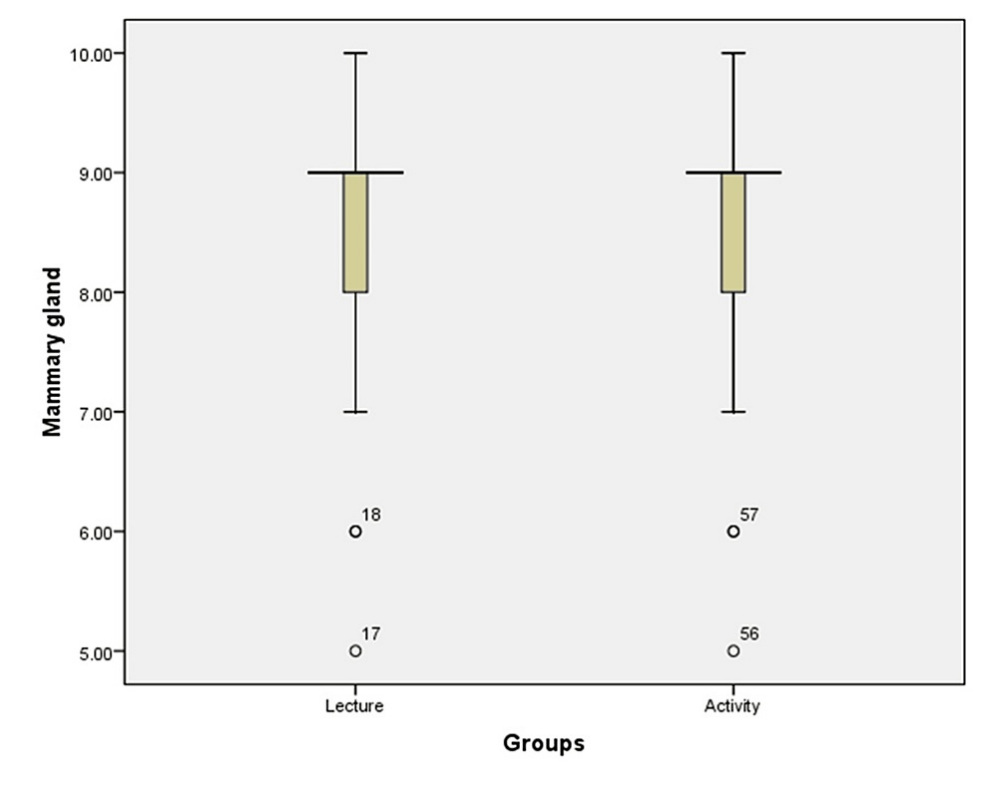
Box graph showing the comparison between the marks scored for the topic mammary gland taught by didactic lecture and activity-based learning method.

Whereas the mean of the scores of the post-test obtained by students who attended a didactic lecture on mammary gland was (8.46), slightly lower than the mean of the scores of the post-test of students who attended activity-based learning on mammary gland (8.60), but the p-value obtained was not significant (0.520) (Table 2) (Figure 3).

**Table 2:** Comparison between the post-test marks of two teaching and learning methods for the topic mammary gland TLM: Teaching/learning materials

TLM	Mean ± SD
Lecture	8.46 ± 1.20
Activity	8.60 ± 1.16
Total	8.53 ±1.18
p-value	p-value=0.520

Since there is no significant difference in the p-values obtained in the above two arms, the hypothesis cannot be rejected.

## Discussion

The field of medical education is continually evolving, with new teaching methods emerging to improve student learning outcomes. The new competency-based medical education curriculum has decreased the duration of didactic lectures to less than one-third of the total duration. So, this study aimed to compare the effectiveness of activity-based learning and didactic lectures in teaching anatomy to MBBS first-year students and determine if activity-based learning alone can be an effective teaching-learning method.

The findings emphasized that didactic lectures were equally effective in enhancing students' retention and understanding of anatomy compared to activity-based learning. The traditional lecture does not foster effective communication and engagement between teachers and students. Activity-based learning encourages active participation and engagement, which can result in a better comprehension of the topic when conducted following a well-structured lecture on the same subject, as well as an opportunity to interact with peers. Furthermore, the hands-on approach of activity-based learning allowed students to explore and discover information for themselves, leading to a deeper and more holistic understanding of the subject. It is also affected by the learning styles and types of each student [7]. However, further research is needed to determine the long-term effects of these teaching methods and to identify ways to improve their effectiveness.

Teachers can improve their professional relationships and enhance their expertise through receiving peer reviews, feedback, and student reflections [8-10]. Implementing this teaching-learning method might prove challenging due to the limited number of faculty available. The current trends in education are shifting from a teacher-centered to a learner-centered approach. Teachers act as facilitators, guiding and supporting students. This transformation is crucial for preparing learners for success in an ever-changing world [11,12].

Didactic lectures were found to be less effective in promoting student engagement and active learning. In academic classrooms, the contributing factor to the lack of student motivation is the lack of inclusion of fun [13]. Lectures can be passive and unengaging, leading to reduced retention of information and limited critical thinking skills. However, lectures can still be an effective teaching method, particularly when supplemented with other teaching methods, such as interactive discussions, visual aids, or activities. It has been shown in numerous works of literature that traditional teaching methods often fail to adequately support students who struggle academically [14].

Activity-based teaching is more effective for the development of higher-order thinking skills in the students [15,16].

The problem-based medical curriculum plays a role in enhancing the professional skills of medical students [17]. It also fosters more generalized work-related skills that are crucial for succeeding in a professional setting [18].

In this study, we found no significant difference between the two methods as the p-value obtained was more than .05, whereas S. Rajeswarie et al., in their study, compared team-based learning over conventional didactic lecture and found that the academic performance of the students was better for team-based learning as compared to those who had undergone conventional didactic lecture. The p-value obtained was also statistically significant for the team-based learning method [19].

Bhavsar et al., in their study, compared a flipped classroom with a traditional didactic classroom and reported that the mean score of students who learned through the flipped classroom method was higher as compared to the traditional didactic classroom method and found a statistically significant result (p<0.05) for each module in the flipped classroom method of teaching and learning [20].

In the present, students had mixed feedback regarding their preferred method of learning. While some were in favor of traditional didactic lectures, the majority preferred a combination of didactic lectures and activity-based learning. This approach was deemed more effective for conceptual understanding of topics compared to either method alone.

According to another study, the flipped classroom method is advantageous over the traditional didactic classroom method in learning, where a large chunk of students felt that the flipped classroom method was time-consuming and difficult [21]. This may be due to group discussion being an integral part of the flipped classroom method, which may bring mental pressure along with it, as reported in other studies.

Anatomy, being a vast and complex subject, requires a lot of time and resources to learn thoroughly. Certain topics of anatomy may be better suited for didactic lectures, while others may be more effectively taught through activity-based teaching. Choosing appropriate topics in anatomy for didactic lectures and activity-based teaching could save time and resources. The choice of teaching method should depend on the specific learning objectives and needs of the students. Educators can focus their efforts on teaching topics more effectively by dividing the topics according to their relevance.

Improved infrastructure, including adequate seating arrangements, various teaching aids like projectors, blackboards, mics, dissection tables, and cadavers, as well as knowledgeable resource persons during activity-based learning, would greatly improve the execution of the task and enhance the effectiveness of the teaching-learning process.

Limitations of the study

Conduction of both didactic lectures and activities on the same topic at the same time was difficult due to time constraints. Activity-based learning can be more time-consuming and resource-intensive, requiring careful planning and coordination to implement effectively. Various topics that cannot be explained through lectures or cannot be visualized through cadaveric dissections can be explained better through activity, and vice-versa. Implementing this teaching and learning approach in large study subjects may prove difficult due to the limited availability of faculty.

## Conclusions

The study highlights the potential benefits of different teaching-learning methods in the subject of anatomy. Activity-based learning can be an effective teaching method for MBBS first-year students. This approach can enhance students’ learning outcomes, promote active participation and engagement, and provide a more holistic understanding of the subject when preceded by traditional lecture. However, it is important to carefully consider the resources, planning, and coordination required to implement activity-based learning effectively and to supplement traditional teaching methods, such as didactic lectures, with other interactive teaching techniques to provide a well-rounded educational experience. This study thus provides valuable insights into the use of innovative teaching methods in medical education. These findings have implications for medical educators and curriculum designers who are seeking to improve student learning outcomes and engagement in medical education.

A few topics in anatomy, like the formation of spinal nerves or synapses, cannot be demonstrated even in dissection or histological examination. For such topics, the activity-based teaching and learning method could be a boon as it helps in the visualization of concepts and better understanding. Topics that are more knowledge-based and do not require much higher-order thinking could be sufficiently taught by didactic lectures. This would help in the better placement of competencies based on its objectives and in efficient time management so that the syllabus could be completed on time.
